# Artificial Intelligence in the Management of Patients with Respiratory Failure Requiring Mechanical Ventilation: A Scoping Review

**DOI:** 10.3390/jcm13247535

**Published:** 2024-12-11

**Authors:** Dmitriy Viderman, Ainur Ayazbay, Bakhtiyar Kalzhan, Symbat Bayakhmetova, Meiram Tungushpayev, Yerkin Abdildin

**Affiliations:** 1 Department of Surgery, School of Medicine, Nazarbayev University, 010000 Astana, Kazakhstan; 2 Department of Anesthesiology, Intensive Care, and Pain Medicine, National Research Oncology Center, 010000 Astana, Kazakhstan; 3 Department of Computer Science, School of Engineering and Digital Sciences, Nazarbayev University, 010000 Astana, Kazakhstan; 4 Department of Mechanical and Aerospace Engineering, School of Engineering and Digital Sciences, Nazarbayev University, 010000 Astana, Kazakhstanyerkin.abdildin@nu.edu.kz (Y.A.)

**Keywords:** machine and deep learning methods, respiratory failure, medical outcomes, intubation, prediction, classification

## Abstract

**Background:** Mechanical ventilation (MV) is one of the most frequently used organ replacement modalities in the intensive care unit (ICU). Artificial intelligence (AI) presents substantial potential in optimizing mechanical ventilation management. The utility of AI in MV lies in its ability to harness extensive data from electronic monitoring systems, facilitating personalized care tailored to individual patient needs. This scoping review aimed to consolidate and evaluate the existing evidence for the application of AI in managing respiratory failure among patients necessitating MV. **Methods**: The literature search was conducted in PubMed, Scopus, and the Cochrane Library. Studies investigating the utilization of AI in patients undergoing MV, including observational and randomized controlled trials, were selected. **Results**: Overall, 152 articles were screened, and 37 were included in the analysis. We categorized the goals of AI in the included studies into the following groups: (1) prediction of requirement in MV; (2) prediction of outcomes in MV; (3) prediction of weaning from MV; (4) prediction of hypoxemia after extubation; (5) prediction models for MV–associated severe acute kidney injury; (6) identification of long-term outcomes after prolonged MV; (7) prediction of survival. **Conclusions**: AI has been studied in a wide variety of patients with respiratory failure requiring MV. Common applications of AI in MV included the assessment of the performance of ML for mortality prediction in patients with respiratory failure, prediction and identification of the most appropriate time for extubation, detection of patient-ventilator asynchrony, ineffective expiration, and the prediction of the severity of the respiratory failure.

## 1. Introduction

Mechanical ventilation is one of the most frequently used organ replacement modalities in the intensive care unit (ICU). It is an unreplaceable element required for saving the lives of patients with acute respiratory disorders. Invasive mechanical ventilation is required in roughly 40% of ICU patients [1]. It should be noted that the maintenance of mechanical ventilation is costly. In the UK and the US, around $2300 is spent daily per patient for ICU stays, covering resource utilization during the initial stay, including assessments of organ support, respiratory assistance, renal replacement therapy, X-rays, chest drains, and medication administration [2,3].

Weaning a patient from MV and facilitating extubation can present challenges, particularly in patients with pulmonary indications for MV, and often requires a significant portion of overall ventilation time. The decision if a patient is ready for extubation mostly depends on physiological functioning, vital signs, consciousness, hemodynamics, respiratory function, and ventilation parameters [4,5,6,7,8].

Conversely, premature extubation has a negative impact. It might lead to a condition known as extubation failure, which is characterized by the inability of a patient to maintain breathing after the extubation. Extubation failure was found to occur in up to 25% of cases [9]. Extubation failure, defined as reintubation within 72 h, was linked to prolonged hospitalization, and extended ICU stays. Complications associated with reintubation, such as prolonged sedation or mechanical ventilation, notably ventilator-associated pneumonia, could further contribute to an extended length of stay [10]. Prolonged ICU stays also deplete economic and medical resources [10,11].

AI has become increasingly integrated into medicine, serving as an additional tool that aids medical personnel in therapeutic and diagnostic decision-making [12]. It has proven to be an efficient decision-making supplement in acute and critical medicine [13]. AI utilizes computational power to solve and find patterns in complex systems. It can analyze an enormous amount of data and produce outcomes in a short time, even data in an image format can be processed. The Komorowski study demonstrated that a specially tailored AI clinician could propose more effective treatment regimens for sepsis, resulting in reduced mortality rates among patients who followed AI-recommended dosages. Sepsis stands as a prominent contributor to hospital mortality [14]. An AI-assisted model (dashboard) helped the respiratory care members predict the optimal time of weaning from MV, shortening the intubation time [12,13,14,15]. Recently, machine learning algorithms have been implemented in the treatment of COVID-19 [16,17,18].

The study highlighted the importance of transparent data and code sharing, along with adherence to reporting standards, to enhance the reproducibility and applicability of AI models in healthcare [19]. There are significant differences among ventilatory systems in terms of their intended applications. While some systems are designed to address specific respiratory conditions, such as acute respiratory distress syndrome (ARDS), or to optimize settings for select respiratory parameters, others target the weaning process in modes such as synchronized intermittent mandatory ventilation (SIMV) or pressure support (PS). Additionally, certain systems aim to optimize a wider range of respiratory parameters across various patient groups and ventilatory modes. AI has the potential to predict changes in MV settings, assisting clinicians in monitoring and adjusting parameters based on the patient’s clinical status, thereby alleviating the burden on healthcare providers in intensive care units (ICUs) and potentially improving treatment strategies for critically ill patients [20].

AI is becoming a promising tool in the management of artificial lung ventilation due to its ability to predict key parameters such as vital capacity, CROP (Compliance-Rate-Oxygenation-Pressure index), Maximal Inspiratory Pressure (MIP), respiratory rate, Airway Occlusion Pressure (P0.1), and Rapid Shallow Breathing Index (RSBI) [20]. However, modern AI-based systems mostly perform predictive tasks, such as forecasting the need for intubation, identifying potential respiratory issues, and assessing readiness and success for weaning [21], without providing real-time adaptation of ventilator settings. This limitation is due to a number of technological and physiological factors, including limited access to codes and patient data [19], and determining the appropriate positive end-expiratory pressure (PEEP) for each individual patient [22]. To overcome these barriers, developments combining monitoring, forecasting, and therapeutic correction in real time are needed.

At the moment, AI functions more like a diagnostic tool that provides the specialist with important data for analysis, but it is not able to independently adjust ventilation parameters. Predictive models are similar to complex monitors that help interpret data, but decision-making remains with the clinician [23]. The implementation of adaptive management requires the creation of integrated decision support systems that are able to learn from patient data, take into account changes in physiological conditions, and adjust ventilation settings according to individual needs.

Existing ventilation methods, including volume-controlled ventilation (VCV) or pressure-controlled ventilation (PCV) are not able to completely eliminate the main physiological problems that arise in patients undergoing artificial lung ventilation [24]. These limitations raise the question of the clinical effectiveness of using AI as an automated tool for setting ventilation parameters. As a result, the use of AI as an automated tool for setting ventilation parameters at this stage of technology development will have an uncertain clinical effect. Without a deeper understanding of physiological reactions and the development of integrated adaptive models, the effectiveness of such solutions remains questionable.

Although the machine learning (ML) methods were found useful in the management of MV in ICU [25] and the prediction of mortality [26], there was no scoping review on the application of AI methods for the management of MV describing the medical tasks and AI methods used for them as well as best methods for each task. AI can be helpful for personnel including anesthesiologists, intensivists, radiologists, pulmonologists, respiratory therapists, and nurses working with patients requiring MV. The objective of this review was to synthesize the available evidence regarding the potential application of AI in MV in patients with respiratory failure.

## 2. Materials and Methods

The current review was conducted according to the PRISMA guidelines for scoping reviews [27]. We considered studies focused on the application of AI for the management of critically ill patients requiring MV.

We considered the following study inclusion criteria:

- Focus on the application of AI in the management of patients with acute respiratory failure requiring MV (including prediction of intubation and MV, selecting the mode of MV, prediction of complications in mechanically ventilated patients, and timing of extubation);
- Study design: observational (prospective, retrospective), randomized controlled trials;
- Language: English.

Exclusion criteria comprised studies with insufficient detail on AI methodologies or applications, lacking relevance to the specific objectives of the scoping review, demonstrating inadequate methodological quality or risk of bias, or consisting of review articles, editorials, letters, or commentaries devoid of original data or analysis.

### Literature Search

For the literature search, PubMed, Scopus, and the Cochrane Library were used. The search was narrowed with the help of search filters to find the most relevant articles.

Search terms: (“respiration, artificial” OR (“respiration” AND “artificial”) OR “artificial respiration” OR (“mechanical” AND “ventilation”) OR “mechanical ventilation”) AND (“artificial intelligence” OR (“artificial” AND “intelligence”) OR “artificial intelligence”)

((((respiratory failure) AND (mechanical ventilation)) OR (artificial intelligence)) OR (machine learning)) OR (deep learning) Sort by: Most Recent ((“respiratory insufficiency” OR (“respiratory” AND “insufficiency”) OR “respiratory insufficiency” OR (“respiratory” AND “failure”) OR “respiratory failure”) AND (“respiration, artificial” OR (“respiration” AND “artificial”) OR “artificial respiration” OR (“mechanical” AND “ventilation”) OR “mechanical ventilation”)) OR (“artificial intelligence” OR (“artificial” AND “intelligence”) OR “artificial intelligence”) OR (“machine learning” OR (“machine” AND “learning”) OR “machine learning”) OR (“deep learning” OR (“deep”AND “learning”) OR “deep learning”). The search details can be found in the Supplementary File.

The final search collection included articles published in English up to April 2023. Despite the initial intention to include multiple languages to capture diverse perspectives, it became apparent that most of the relevant literature fitting the study’s scope and criteria was primarily available in English. Moreover, the decision to limit the search to English articles was influenced by the predominance of English as the language of scientific communication and the desire to maximize accessibility, relevance, and impact within the academic community. The search terms can be found in the Supplementary File.

A further selection of articles was conducted manually by reading the title and abstract. Articles that did not match the scope of the review were excluded. The following data were extracted: author, year, mean age, sample size, age, outcomes, adverse events, limitations, and conclusions. Two reviewers conducted sequential evaluations of the titles, abstracts, and full texts of all publications identified through our searches to identify potentially relevant publications. Disagreements on study selection and data extraction were resolved through consensus and discussion with other reviewers if necessary.

## 3. Results

**Figure 1:**
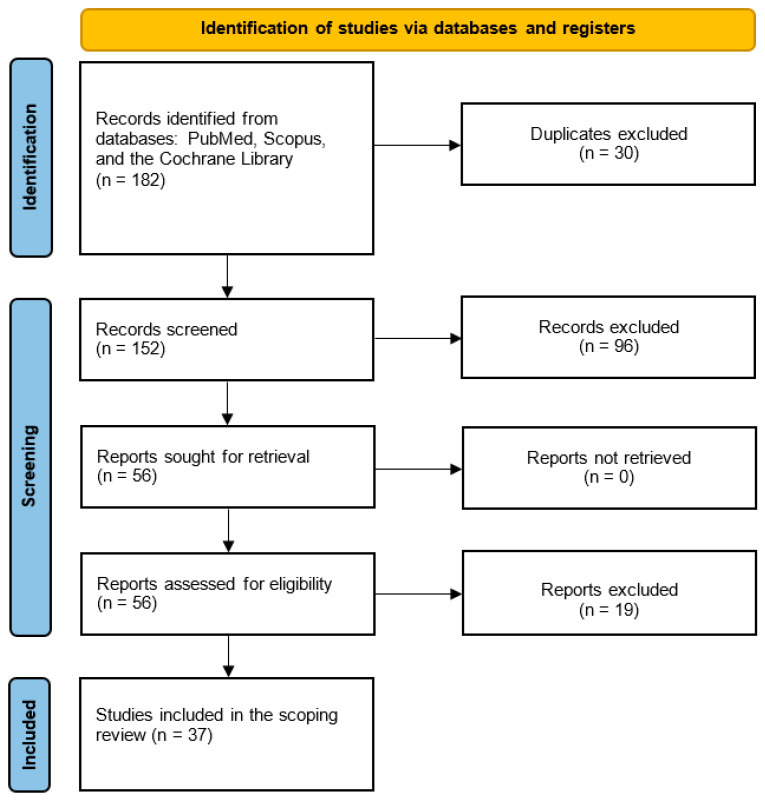
PRISMA diagram.

Overall, 182 articles were initially identified, of which 30 duplicates were excluded. Of the 152 screened articles (artificial intelligence in mechanical ventilation—56; artificial intelligence in artificial ventilation—58, machine learning in mechanical ventilation—34, deep learning in mechanical ventilation—4), 96 were additionally excluded. After careful review and assessment for eligibility, 19 articles were removed and 37 articles were finally included in the analysis (Figure 1, Supplementary file—Tables S1 and S2) [12,15,28,29,30,31,32,33,34,35,36,37,38,39,40,41,42,43,44,45,46,47,48,49,50,51,52,53,54,55,56,57,58,59,60,61,62].

We categorized the goals of AI in the included studies into the following groups: (1) prediction of requirement in MV (upon patient admission); (2) prediction of outcomes in mechanically ventilated patients; (3) prediction of weaning from MV and extubation; (4) prediction of MV mode; (5) prediction of hypoxemia after extubation; (6) prediction models for MV-associated severe acute kidney injury; (7) identification of long-term morbidities and outcomes after prolonged MV; (8) prediction of survival following ICU admission; (9) prediction of MV support pressures; (10) decision support for mechanical ventilation and prevention of complications; (11) implemented AI algorithms, its advantages, limitations, and performance evaluation.

### 3.1. Patient Characteristics

The included studies enrolled various groups of critically ill patients with the following diseases, co-morbidities, and syndromes [12,15,28,29,30,31,32,33,34,35,36,37,38,39,40,41,42,43,44,45,46,47,48,49,50,51,52,53,54,55,56,57,58,59,60,61,62]:

1. Respiratory system: pneumonia, post-operative respiratory failure, chronic bronchitis, asthma, emphysema, acute respiratory distress syndrome, COVID-19 pneumonia, pulmonary edema, asthma, pneumonia;
2. Cardiovascular system: hypertension, arrhythmia, pulmonary vascular diseases, chronic heart failure, acute myocardial infarction, septic shock, hemorrhagic shock, cardiac arrest;
3. Neurological system: coma, cerebral vascular disease, and dementia;
4. Gastro-intestinal system: peritonitis, pancreatitis, liver cirrhosis, hepatitis;
5. Renal system: chronic kidney failure;
6. Genito-urinary system: urinary tract infections;
7. Poisonings: carbon monoxide and other toxicities;
8. Miscellaneous: critically ill COVID-19 patients, diabetes mellitus, malignant neoplasm, AIDS/HIV, obesity, diabetes, rheumatologic disorders.

### 3.2. Characteristics of Included Studies

#### 3.2.1. Prediction of the Requirement in MV

Tracheal intubation and initiation of MV is a critical decision that can affect outcomes in patients with respiratory failure. The studies included in this category reported that AI might be useful for decision-making and resource allocation [29,30,31,39,42]. The prediction models demonstrated good predictive capacity before intubation and initiation of mechanical ventilation (up to 24 h), helping in the decision-making. The models require external validation and further improvement of their performance on different patient populations, and clinical settings.

#### 3.2.2. Prediction of Outcomes in Mechanically Ventilated Patients

We can highlight the following studies, which focused on the prediction of outcomes in mechanically ventilated patients with respiratory failure.

Gourdeau et al. developed an automated machine-learning algorithm that uses bedside chest X-rays to provide a predictive probability of mortality following mechanical ventilation [40]. The cohort of the study was ICU-admitted COVID-19 adult patients, and the CheXpert dataset of 224,316 X-ray images was used. The dataset included 224,316 X-ray images from 65,240 patients at Stanford Hospital, used to train a CXR-specific feature extractor. The evaluation employed a curated validation set of 234 images from the CheXpert dataset. To ensure label reliability, three board-certified radiologists from the Stanford CheXpert team individually assigned findings and diagnoses to each study in this validation set. The predicting model was trained with extracted features and showed 0.702 (95% CI: 0.707, 0.694) AUC. Then, a combination of imaging results and other risk factors increased the AUC to 0.743 (95% CI: 0.746, 0.732). Moreover, the authors suggest that using high-quality chest X-ray images increases the prediction accuracy and gives even stronger results.

The study by Bermejo-Peláez focuses on developing an AI system utilizing a deep learning algorithm to primarily predict the need for mechanical ventilation (MV) as the primary outcome among patients with COVID-19 pneumonia using their CT scans, with secondary comparisons to assessments by human readers [45]. The model assessed a sample size of 103 patients, examining them for the presence of various types of lung lesions associated with COVID-19, including ground glass opacities and consolidation. These lesions were manually annotated by a radiologist on CT images obtained from a database provided by the Italian Society of Medical and Interventional Radiology. As a result, the agreement between models’ and radiologists’ assessments of parenchymal damage was strongly correlated (Spearman correlation coefficient 0.83). The AUC of prediction of mortality probability, ICU admission, and MV requirement were 0.87 (95% CI: 0.790, 0.959), 0.73 (95% CI: 0.582, 0.871), and 0.68 (95% CI: 0.496, 0.862), respectively. The authors reported that the AI model showed a better prediction of clinical outcomes compared to radiologists’ outcome prediction and suggested a better prognosis for patients.

#### 3.2.3. Prediction of Hypoxemia After Extubation

Xia et al. conducted a study to analyze six different machine-learning algorithms for the precise prediction of hypoxemia after extubation for patients older than 18 years of age in the ICU [47]. They split the dataset into training (80%) and final (20%) sets. According to the training, the RF (random forest) and LightGBM were the strongest. It was found that 1877 out of 14,777 patients had hypoxia after the extubation. The area under the curve of RF was 0.792 (95% CI: 0.771, 0.814), and 0.792 (95% CI: 0.770, 0.815) according to LightGBM, which suggested that the machine learning models have significant potential in hypoxia prediction.

#### 3.2.4. Prediction Models for MV-Associated Severe Acute Kidney Injury

According to Huang et al., the developed machine-learning models may be effective in facilitating the early detection of acute kidney injury (stages AKI-23, AKI-3) associated with mechanical ventilation [48]. Random forest and logistic regression algorithms were chosen, and the RF outperformed the second. The incidences of AKI-23 were 154 and 119 in internal and external validation cohorts, with areas under the curves 0.78 (95% CI: 0.74, 0.82) and 0.80 (95% CI: 0.76, 0.84). In addition, 81 and 67 patients had AKI-3 with areas of 0.81 (95% CI: 0.76, 0.87) and 0.80 (95% CI: 0.73, 0.86).

#### 3.2.5. Identification of Long-Term Morbidities and Outcomes After Prolonged MV

Maddux et al. aimed to identify long-term morbidities and associated risk factors after discharge using unsupervised machine learning techniques [49]. The subjects were 381 pediatric patients with a median age of 3.3 years, and 147 of them had complex chronic conditions such as respiratory, injury, and neurologic conditions. Scientists suggested three phenotypes: lower morbidity, higher morbidity, and 1-year non-survivors. Longer ICU stays and tracheostomy lead to higher morbidity, chronic conditions to non-survivor phenotypes. In conclusion, longer mechanical ventilation stays predicted new morbidities, and identifying the phenotypes may help with prognosis.

### 3.3. Classifications by Tasks and Methods

**Figure 2:**
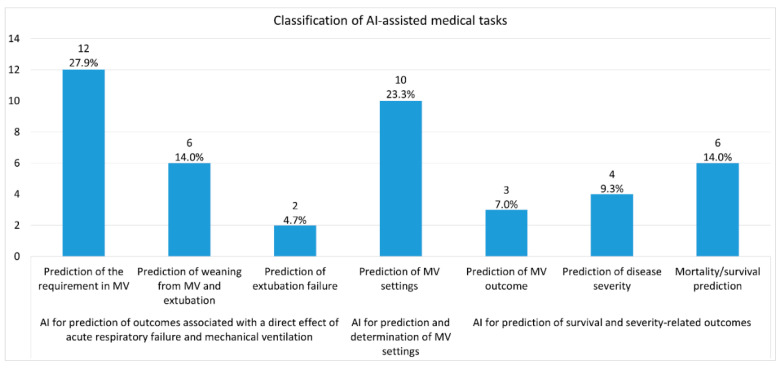
Classification of AI-assisted medical tasks.

The 37 papers included in the meta-analysis addressed seven medical tasks (Figure 2) related to mechanical ventilation and used 12 different AI methods to address them. The studied patients are adults, and their comorbidities were considered in the studies. Their primary diagnoses were COVID-19 and other lung diseases. All the proposed AI models were successful with an average accuracy of about 80%. We now propose the following classifications.

Classification of AI-assisted medical tasks

1. Prediction of the requirement in MV
2. Prediction of weaning from MV and extubation
3. Prediction of extubation failure
4. Prediction of MV settings
5. Prediction of MV outcome
6. Prediction of disease severity
7. Mortality/survival prediction

The first three generally represent AI for the prediction of outcomes associated with a direct effect of acute respiratory failure and mechanical ventilation, the fourth task represents AI for the prediction and determination of MV settings, and the last three represent AI for the prediction of survival and severity-related outcomes. Twelve out of 37 studies (27.9%) studied the prediction of the requirement in MV and 10 (23.3%) studied the prediction of MV settings; these were the two most popular tasks.

#### 3.3.1. Classification of AI Methods

**Figure 3:**
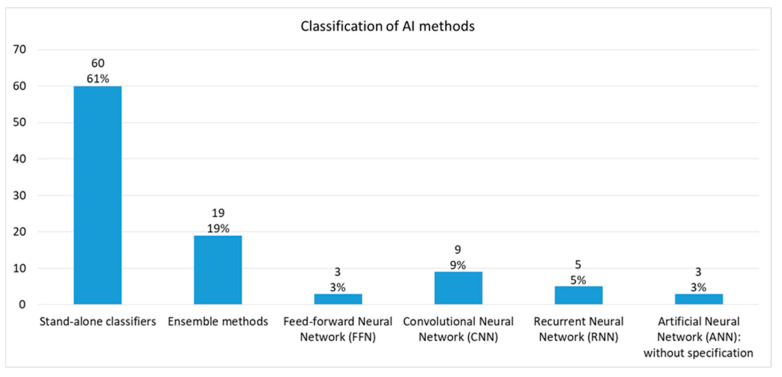
Classification of AI methods.

Twelve AI methods (Figure 3) used in 37 studies included both DL (namely, feed-forward neural networks, convolutional, recurrent, and artificial neural networks) and non-DL methods (Stand-alone classifiers and ensemble methods). Among the 60 stand-alone classifiers used in the studies, logistic regression and random forest methods were used the most—19 and 17, respectively, followed by SVM (10), k-nearest neighbors (6), and decision tree (2). Gradient boosted decision tree (GBDT) was the most used method (13) in ensemble methods followed by gradient boosting machine (6).

#### 3.3.2. Medical Tasks and AI Methods Used for Them

**Figure 4:**
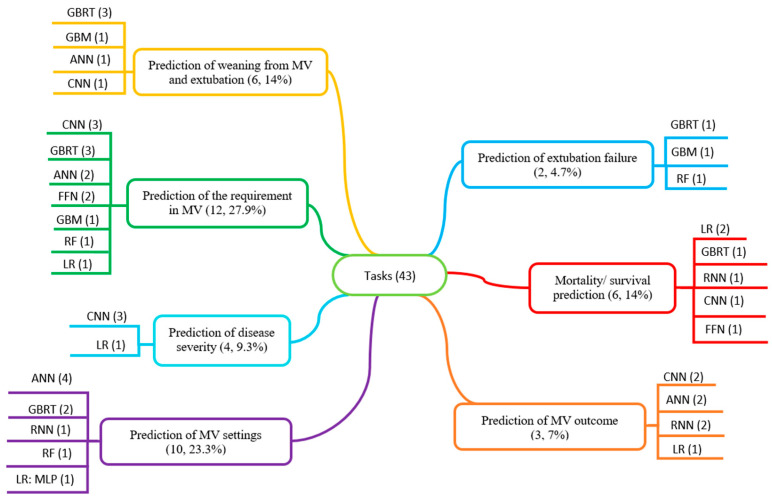
Medical tasks and AI methods used for them (43 medical tasks from 37 studies were classified into seven prediction categories with numbers and percentages of tasks in each category; AI methods and the number of studies used them are shown for each category).

A total of 37 studies aimed to solve seven different medical tasks using various AI methods (Figure 4). Listed below are the AI methods used for each of the tasks and those that showed the best performance based on the criteria used in the studies.

1. For the prediction of the requirement in MV different methods like FFN, CNN, ANN, GBM, GBRT, RF, and LR were used in 12 studies. The most used methods were CNN and GBRT—each used in three studies. The best method was FFN, which was used in two studies and achieved an AUC value of 0.99 (reported by Aslam, 2022 [37]).
2. There are six articles for the purpose of predicting weaning from MV and extubation using different methods like CNN, ANN, GBRT, and GBM. Although the GBRT model was presented as the best method in three studies, CNN outperformed it with an AUC value of 0.94 (reported by Jia et al., 2021 [33]).
3. Methods such as GBM, GBRT, and RF were used to predict extubation failure in two articles. The best method was the CatBoost model (GBRT), which had a higher AUC value of 0.84 (reported in Zhao et al., 2021) compared to other methods (LightGBM, RF) which had a value of 0.792 (reported by Xia, 2022 [47]).
4. For the prediction of MV settings methods like ANN, CNN, RNN, LR, RF, and GBRT were used. The most-used method was ANN, which was used in four studies. The best performance was shown by a convolutional network with a long short-term memory (combination of CNN and LSTM) with an accuracy value of 99% (reported by Chen et al., 2022 [43]).
5. In three studies, methods such as CNN, RNN, ANN, and LR were used to predict the MV outcome. The best performance was shown by a convolutional long short-term memory network (combination of CNN and LSTM) with an accuracy value of 99% (reported by Chen et al., 2022 [43]).
6. Methods such as CNN and LR were used to predict disease severity. Three out of four articles used CNN models; their AUC value was higher than LR, 0.87, with the highest value being 0.929 (reported by Han et al., 2022 [35]).
7. There are six articles for the purpose of predicting mortality/survival using different methods such as CNN, RNN, FFN, LR, and GBRT. Among them, the FFN model showed the best results with an outstanding AUC value of 0.998 (reported by Aslam, 2022 [37]).

## 4. Discussion

MV is crucial for respiratory support in patients with respiratory failure. However, improper settings may result in severe complications, exacerbating morbidity and mortality. AI might be potentially helpful in various tasks, such as patient risk identification, with the utilization of these techniques possibly leading to improved outcomes via early intervention opportunities.

In this scoping review, seven medical tasks were identified where AI demonstrated promising performance. Namely, the FFN method was best for predicting the requirement for mechanical ventilation (MV), while also demonstrating superior accuracy in predicting mortality/survival. The CNN method exhibited excellent performance in predicting both weaning from MV and extubation, alongside demonstrating proficiency in predicting disease severity. The CatBoost model outperformed others in predicting extubation failure, while for predicting MV settings and outcomes, the combination of CNN and LSTM showed the best performance.

Building on the insights gained from our scoping review regarding the promising performance of AI in various medical tasks, it is evident that AI has substantially transformed the landscape of medicine and healthcare in recent years. This transformation is further evidenced by the widespread adoption of AI technologies in clinical settings, where they have been used to reveal information from clinical datasets and assist clinicians in a wide variety of tasks including facilitation with screening, diagnosis, triage, risk analysis, and administrative operations [63,64,65]. AI technologies, including the global AI market in healthcare, has the potential to grow more than 10 times in the next few years [66].

However, despite the rapid research and development of various AI models, the implementation and use of AI in clinical practice are limited [63,64,65]. The research–practice gap in AI appears to be wide. There have been no advances in ‘device into practice’ technology, even though ‘algorithm into model’ research has intensified in the last few years [19]. The development, and especially the application, of AI algorithms frequently suffers from several technical issues including methodological issues, bias, overfitting, and low generalizability [66], which limit the safe transition of the AI models from research into clinical care [67].

Most of the currently available evidence and guidelines are predominantly based on population data and might not apply to single patients. There is not enough experimental data to personalize ventilation [68]. Moreover, it is difficult for intensivists and respiratory therapists to continuously monitor dynamically changing respiratory mechanics and adjust the settings of ventilation. Therefore, AI could be a solution for achieving a more personalized approach to mechanical ventilation, predicting respiratory failure and requirements in ML, selecting the most appropriate settings of MV, and forecasting the outcomes.

AI was useful in decision-making and diagnosis in patients with respiratory disorders. It is summarized that machine learning and deep learning methods perform better than traditional and statistical methods [69]. This is explained by their ability to handle missing data and better algorithm structure [70,71]. Moreover, some of the models were hybrid. Even though high prediction values were reported in some articles, AI models are not perfect and have their limitations. When presented with an excessive number of input variables, optimization becomes essential to minimize the input count. Collecting many variables can be cumbersome and time-consuming in practice, potentially leading to increased mortality rates. The performance of AI models has yet to be enhanced to make more reliable prediction conclusions.

Limitations: the scope of this review is broad and covers all aspects of artificial intelligence in mechanical ventilation. Additionally, the preponderance of patients with COVID-19 and primary pulmonary etiologies in the included studies suggest that the findings may not apply to other patients with non-pulmonary pathology as the primary reason for mechanical ventilation. The main goals, reporting style, and assessment of the model performance of the included studies were heterogeneous. Included studies reported different performance metrics: sensitivity and specificity, and positive/negative predictive values, whereas others reported areas under the curve. Most of the studies were observational (retrospective); therefore, the performance of these models might be different in randomized controlled studies.

### Future Directions

While the currently available models exhibit promising performance, further research is essential to ascertain the clinical applicability of AI models and their effectiveness and safety in enhancing respiratory care quality.

However, since most of the developed AI applications mostly focused on prediction, the future direction might be to develop models that not only predict outcomes but also provide MV parameters based on available data, especially if the algorithm could suggest the setting according to the personal needs of a particular patient. Incorporating additional parameters, such as comorbidities and internal organ function disorders could improve the precision of predictions. Additionally, these models should be tested in randomized controlled trials and then possibly even in the format of implementation studies because even if a model was developed and tested, even in the format of randomized controlled trials, in one patient population, it might not be equally suitable in another population.

The primary objective of these models is to improve patient outcomes and streamline medical professionals’ tasks. However, a crucial question remains: will these advancements translate into real-world benefits for patients? Answering this question will shape future research and clinical applications, guiding efforts to ensure that AI innovations effectively contribute to enhanced healthcare delivery and patient care.

## 5. Conclusions

Based on reviewed articles on AI in mechanical ventilation, several points can be drawn. The introduction of additional parameters such as comorbidities and internal organ function disorders improves the accuracy of predictions. When too many input variables are given, optimization is necessary to reduce the number of inputs. A high number of variables is tedious to collect which may be troublesome and time-consuming in practice. Therefore, the mortality rate may rise. Standardization of medical databases is needed, especially for commercial AI algorithms, since they are not open source and modification of the code is not possible. Not all models can handle missing information in the database and still produce results. For instance, in MEWS, missing data cannot be processed at all. Incomplete data may affect prediction accuracy. Even if a control system was proven to perform as well as a human control, it still requires human supervision on spontaneous changes and adjustments. Integration of an AI algorithm with features, i.e., counterfactual explanations and feature importance, enhances the trustworthiness and prediction accuracy of the algorithm. AI has been studied in a wide variety of patients with respiratory failure requiring MV. Comparable with the application of AI in other areas of medicine, the application of AI in the included studies involved prediction task. Common applications of AI in MV included the assessment of the performance of ML for mortality prediction in patients with respiratory failure, the prediction of respiratory failure and requirement in MV at admission, the prediction and identification of the most appropriate time for extubation, the detection of patient–ventilator asynchrony, ineffective expiration, the prediction of the severity of the respiratory failure, the prediction of clinical outcomes in patients with respiratory failure, the prediction of hypoxemia after extubation, the prediction of mechanical ventilation-associated severe acute kidney injury, the prediction of long-term morbidities and outcomes after prolonged MV, the prediction of survival in mechanically ventilated patients with respiratory failure. The performance of AI in ML was reported as high with the potential to improve the management of mechanically ventilated patients with respiratory failure.

## Data Availability

Not applicable.

## Supplementary Materials

The following supporting information can be downloaded at: https://www.mdpi.com/article/10.3390/jcm13247535/s1, Table S1: Study and cohort information; Table S2: Artificial intelligence method and its purpose.

## Abbreviations

ACP	clinical AI predictor
AFib	atrial fibrillation
AI	artificial intelligence
AIDS	acquired immune deficiency syndrome
AIP	imaging AI predictor
ANN	artificial neural network
ARDS	acute respiratory distress syndrome
AUC	area under the curve
C1AIN	integrated clinical and AI based nomogram
CatBoost	categorical boosting
CHD	coronary heart disease
CHF	congestive heart failure
CKD	chronic kidney disease
CLD	chronic liver disease
CNN	convolutional neural network
COPD	chronic obstructive pulmonary disease
CROP	Compliance-Rate-Oxygenation-Pressure index
CT	computed tomography
CVS	cardiovascular system
CXR	chest X-Ray
DL	deep learning
DM	diabetes mellitus
ESRD	end-stage renal disease
FFBP	feed-forward backpropagation (algorithm)
GBDT	gradient boosting decision tree
GI	gastrointestinal system
HBV/HCV	hepatitis B virus / hepatitis C virus
HIV	human immunodeficiency virus
HTN	hypertension
ICU	intensive care unit
IHD	ischemic heart disease
KNN	k-nearest neighbors
LDA	linear discriminant analysis
LightGBM	light gradient boosting machine
LM	Levenberg–Marquardt (algorithm)
LR	logistic regression
LSTM	long short-term memory
MEWS	modified early warning score
MI	myocardial infarction
MIP	Maximal Inspiratory Pressure
ML	machine learning
MLA	machine learning algorithm
MLF	multilinear fitting
MLP	multilayer perceptron
MV	mechanical ventilation
N.S.	not specified
NN	neural networks
P0.1	Airway Occlusion Pressure
PEEP	positive end expiratory pressure
PCV	pressure ventilation
PICU	pediatric intensive care unit
PS	pressure support
PVD	peripheral vascular disease
QDA	quadratic discriminant analysis
RALE	radiographic assessment of lung edema
RF	random forest
RFE	recursive feature elimination
RNN	recurrent neural network
ROX	ratio of pulse oximetry/FiO2 to respiratory rate
RR	respiration frequency
RSBI	Rapid Shallow Breathing Index
SIMV	synchronized intermittent mandatory ventilation
SMOTE	synthetic minority oversampling technique
SVM	support vector machine
TB	tuberculosis
TRL	technology readiness level
UTI	urinary tract infection
VCV	Volume Controlled Ventilation
VT	tidal volume
XGBoost	extreme gradient boosting
