# Supplementary material for: Artificial Intelligence in the Management of Patients with Respiratory Failure Requiring Mechanical Ventilation: A Scoping Review
Source: J Clin Med. 2024 Dec 11;13(24):7535. doi: 10.3390/jcm13247535 (PMC11728182; doi:10.3390/jcm13247535)
Supplement: Supplementary file 1 [file jcm-13-07535-s001.zip › jcm-3307178-supplementary.pdf]

Table S1. Study and cohort information.

#	Author, country, year	Study goals	Sample size	Primary diagnosis	Diseases/ Comorbidities
Retrospective studies					
1	Mamandipoor et al., Italy/Spain/Austria/Germany/China/France/Argentina/Columbia/Canada/Mexico/Brazil/Uruguay/Australia/India/Turkey/South Korea/Tunisia/Ecuador/Greece/Morocco/Thailand/USA, 2021	Evaluate the performance of ML methods in a multicenter cohort of patients for mortality prediction	12596	Respiratory disorders (Mechanically ventilated critically ill patients)	Respiratory disorders: COPD, Asthma, interstitial lung disorders, ARDS, Pneumonia Additional disorders: kidney, liver, general metabolism, and respiratory disorders
2	Nopour et al., Iran, 2023	To predict intubation risk	482 (needs intubation – 176, doesn't need – 306)	COVID-19	Blood pressure, diabetes, and cardiac, and other underlying diseases
3	Bae et al., USA, 2021	To predict MV use and mortality	691	COVID-19	N. S.
4	Hiremath et al., USA/China, 2021	To predict MV use	869 (D1 – 822, D2 – 47)	COVID-19	N. S
5	Pai et al., Taiwan, 2022	To predict the timing of extubation	5940 (extubated patients – 3657, not extubate - 2283)	Critically ill patients requiring MV	N. S. (Charlson Comorbidity Index was used)
Retrospective cohort studies					
6	Jia et al., UK, 2021	Performance evaluation set – 231 (10%)	2299 Training set – 1839 (80%) Validation set – 229 (10%)	N. S. (Critically ill MV patients in ICUs)	N. S. (Patient states are monitored every hour and indicated when patients are potential candidates for extubation thus prompting clinicians to commence

					Spontaneous Breathing Trials)
7	Han et al., China, 2022	To evaluate disease severity using chest CT	117	COVID-19	HTN, diabetes, CHD
8	Radhakrishnan et al., India, 2022	To predict the level of inspired oxygen, MV mode and PEEP	20	Acute respiratory syndrome	N. S.
9	Liao et al., Taiwan, 2022	To predict the time of weaning from MV	670	Patients needing tracheotomy or endotracheal intubation	Diabetes, COPD, MI, stroke, ESRD, pneumonia, sepsis
10	Aslam, Saudi Arabia, 2022	To predict MV use and death	5739 (1513 for mortality, 1508 for MV)	COVID-19	Diabetes, CKD, asthma, coronary artery disease, HTN, cancer, COPD, liver disease
11	Rees et al., Denmark, 2022	To assess the need for changes in MV parameters	95	ICU population: pneumonia, septic shock, peritonitis, post-operative respiratory failure, coma, cardiac arrest, hemorrhagic shock, pancreatitis	COPD, CKD, HTN, IHD, arrhythmia, CHF, PVD, DM, neurological disease, CLD, cancer
12	Bendavid et al., Israel, 2022	To predict respiratory failure and MV use	11,816 – hypoxemic for training<br>1061 – COVID-19	Hypoxemic.<br>Critically ill COVID-19 patients	-
13	Gourdeau et al., Canada, 2022	To predict MV outcome	161	ICU COVID-19 patients	Chronic cardiac disease, chronic pulmonary disease, asthma, CKD, moderate or severe liver disease, chronic neurological disorder, malignant neoplasm, AIDS/HIV, obesity, diabetes, rheumatologic disorder, dementia, smoker

14	Lin et al., Taiwan, 2021	To predict weaning from MV	963	Cerebral vascular disease, other (unspecified)	HTN, DM, CHF, AFib, COPD, asthma, ESRD, liver cirrhosis, cerebral vascular disease, malignancy
15	Serrano et al., Portugal and Spain, 2021	To predict the use of MV	N. S	Critically ill COVID-19	N. S. Glasgow Coma Scale, Simplified Acute Physiology Score II, Acute Physiology and Chronic Health Evaluation II were provided for the cohort
16	Chen et al., China, 2022	To detect patient-ventilator asynchrony from MV: ineffective expiration, double triggering	10	Chronic bronchitis, asthma, emphysema, ARDS	N. S.
17	Lu et al., China, 2021	To predict the severity of disease in obese patients	213	COVID-19 patients	HTN, DM, cardiovascular disease, cerebrovascular disease, chronic pulmonary disease, hepatitis or liver cirrhosis, chronic renal failure, malignancy
18	Bermejo-Peláez, Spain, 2022	To predict clinical outcomes	103	COVID-19 pneumonia	N. S.
19	Cheng, Taiwan, 2022	To predict MV mode changes	1483	Pulmonary edema, systolic CHF, acute MI, COPD, asthma, pneumonia, broncho-pneumonia, UTI, sepsis, the toxicity of carbon monoxide	DM, brain-related (infarction/ hemorrhage), kidney-related (ESRD, acute/chronic kidney disease), liver-related (alcoholic, HBV/HCV cirrhosis), lung-related (TB), malignancy

20	Xia, China, 2022	To predict hypoxemia after extubation	14777	ICU patients	CHF, HTN, pneumonia, respiratory failure, DM, heart failure, cerebrovascular disease, renal disease, liver disease, cancer
21	Huang, China, 2022	To predict models of mechanical ventilation–associated severe acute kidney injury	Development: 3986<br>Validation: 1210	Sepsis, cardiovascular, other respiratory, neurological, other	HTN, DM, CHF, chronic pulmonary disease, CKD, chronic liver disease
22	Maddux, USA, 2022	To identify long-term morbidities and outcomes after prolonged MV	633	PICU patients: respiratory, injury, neurologic, infectious, GI, oncologic, CVS, poisoning, dermatologic, genetic, transplant	N. S. (comorbidities were used to categorize the patients as having a pre-existing complex chronic condition)
23	Pinto et al., Colombia/Spain, 2023	To analyze respiratory pattern variability	154 (Successful group – 94, failure – 39, re-intubated – 21)	Patients undergoing weaning from MV	N. S.
24	Liu et al., Taiwan, 2022	To predict optimal timing for MV weaning	5873<br>Training dataset – 4111 (70%),<br>testing - 1762 (30%)	Adult ICU patients with invasive MV	N. S.
25	Chan et al., Taiwan, 2022	To predict long-term mortality in critically ill ventilated patients	6994 (survivor – 4589, non-survivor – 2405)	Critically ill patients requiring MV	N. S. (Charlson Comorbidity Index was used)
26	Hagan et al., UK, 2020	To construct a personalized predictive alert system for violation of tidal volume thresholds during the periods of MV	22	Patients in ICU	N. S.

27	Parreco et al., USA, 2018	To identify patients at risk for prolonged MV and tracheostomy placement	20262	Coronary atherosclerosis of native coronary artery, aortic valve disorders	Fluid and electrolyte disorders, COPD, cardiac arrhythmias, congestive heart failure, coagulopathy, other neurological conditions, alcohol abuse, valvular disease, weight loss, pulmonary circulation, paralysis, deficiency anemia, metastatic cancer, depression, peripheral vascular disease, blood loss anemia, diabetes, drug abuse, lymphoma, hypothyroidism, hypertension, liver disease, obesity, renal failure, solid tumor, psychosis, peptic ulcer disease, rheumatoid arthritis, AIDS
Cohort study					
28	Ebrahimian et al., USA/India, 2021	Predict patient outcomes and the need for MV in COVID-19 pneumonia based on performance of AI and RALE scores from CXRs	405<br>Site A – 226<br>Site B – 179	COVID-19 pneumonia	Cardiovascular diseases, kidney diseases, liver diseases, cancer, respiratory diseases, blood disorders, metabolic syndromes, and neurodegenerative disorders
Observational study					
29	Shashikumar et al., USA, 2021	Predict when to utilize MV using a DL model	General development site – 18528	Not provided for general cohort	N. S. However, the Charlson comorbidity

		for hospitalized and Covid-19 positive patients up to 24 h in advance.	General validation site – 3888<br>Covid-19 development site – 26<br>Covid-19 validation site – 402	COVID-19 for COVID-19 cohort	index was provided for each group
Observational prospective study					
30	Ferrari et al., Italy/UK, 2020	Predict a 48-hour respiratory failure in patients with COVID-19 that will require MV	198	COVID-19	Dyspnea, Chronic Kidney Insufficiency were used as additional inputs for models 3 and 4
Observational retrospective study					
31	Kimura-Sandoval et al., Mexico, 2021	To determine the risk of mortality/MV	166	COVID-19	Diabetes, hypertension, COPD, smoking, asthma, obesity, immunosuppression, cardiovascular disease, cancer, organ transplant
Prospective studies					
32	Burdick et al., USA/Belgium, 2020	Predict the need for MV within the next 24 h.	MLA = 197<br>MEWS = 183	COVID-19	Sepsis, ARDS, Pneumonia, Acute Kidney Injury
33	Zhao et al., Australia/China, 2021	To predict extubation failure in ICU	MIMIC-IV dataset – 16189, Zhongshan hospital cohort – 502	Ventilated critically ill patients	N. S.<br>(Charlson Comorbidity Index was used)
34	Perchiazzi et al., Sweden/Italy, 2003	To evaluate whether a technology based on ANN could estimate the static compliance of the respiratory system	24 (pigs)<br>Training set - 16, trial - 8	Pigs with healthy and sick lungs	N. S.
Case study					
35	Tzavaras et al., Greece/UK, 2007	Assess the performance of a developed neuro-fuzzy controller based on non-invasive parameters	N. S.<br>(Training set – 60%, evaluation set – 40%)	COPD	N. S.

		for the determination of respiration frequency and tidal volume values for COPD patients.			
		Secondary analysis of prospective cohort			
36	Greco, Italy, 2022	To predict survival at 7, 14, 21, and 28 days from ICU admission	1484: model A (demographics + comorbidities, testing) 929: model B (demographics, comorbidities, 1st day ICU ventilation parameters)	Critically ill COVID-19	Hyper-cholesterolemia, DM, malignancy, cardiac disease, COPD, CKD, hepatic disease
		Computer simulation			
37	Meena, India, 2022	To predict the pressure of MV	(simulated)	N. S.	N. S.

AI algorithms used in the included studies can be found in Table 2 [12,15,23–57].

Table S2. Artificial intelligence method and its purpose.

Paper #	Author, country, year	AI algorithm, model, or method	Comparator (if any)	Purpose of AI	Benefits of AI	Risks of AI	Study conclusion
			1. Prediction of the requirement in MV				
1	Shashikumar et al., USA, 2021	VentNet	ROX index and logistic regression model	Predict the need for MV up to 24h in advance	The optimization of intubation timing; better allocation of resources; real-time decision-making	No particular risk of the model was described	The VentNet DL model performed better than others and its results were externally validated
2	Ferrari et al., Italy/UK, 2020	Microsoft LightGBM	LR	Predict the need for MV in 48 h	Good at handling missing data, data-driven, and robust	Requires 20 variables that may be difficult to provide case scenarios	The algorithm performed well and was found to be beneficial in prediction

3	Burdick et al., USA/Belgium, 2020	Model based on the XGBoost Classifier	MEWS	Predict the need for MV within the next 24 h	Works in the presence of missing inputs; more accurate, sensitive, and specific	Imbalanced data; multiple hypothesis testing biases	A new MLA outperformed MEWS, and is believed to be helpful in addressing risks associated with Covid-19, mortality, and morbidity
4	Ebrahimian et al., USA/India, 2021	Commercial Qure.ai algorithm	RALE score	Predict the need for MV in COVID-19 pneumonia patients	Quantitative, quick, automated; have the least impact on the CXR interpretation process	Attenuation of radiographic opacities, changes in CXR image quality	AI prediction outcomes were found to be as robust as RALE scores. The result can be improved by introducing additional laboratory and clinical information
5	Aslam, Saudi Arabia, 2022	Model is not described - agnostic. Methods - SMOTE with oversampling and SMOTE with undersampling	-	To predict MV use and death	Big data sample	Some lab data are missing. Vaccination is not considered	The model was good in prediction
6	Bendavid et al., Israel, 2022	XGBoost algorithm	LR, RF, CNN, long short-term memory	To predict respiratory failure and MV use	Training on non-COVID patients	Small dataset. Not all features were reported	The model was good in predicting
7	Serrano et al., Portugal and Spain, 2021	RF, XGBoost, KNN, Stochastic Gradient Descent Classifier, Decision	-	To predict the use of MV	Many models were compared	Many parameters were not reported in paper. Outcomes for	RF was the best model

		Tree Classifier, Support Vector Classification				other models were not reported	
8	Nopour et al., Iran, 2023	FFBP-LM (ANN)	-	To predict the risk of intubation	Can analyze noisy, imbalanced, and incomplete datasets; simple, user-friendly, easy-to-use	Only limitations of datasets and input variables were mentioned	ANN model performed well
9	Bae et al., USA, 2021	U-Net DL model, Radiomics, CNN, LDA, QDA, RF	Radiologist Severity Scores, CNN, CNN and Radiomic-Map Embedding	To predict MV requirement	Validity on a multi-institutional dataset. Many models were compared	Nonuniform data. A limited number of clinical features were studied	The models were successful. The inclusion of radiomic features in DL improves model predictions
10	Hiremath et al., USA/China, 2021	C1AIN (CNN)	AIP, ACP	To predict MV use	Accurate, easy to deploy, hybrid approach	No access to the raw preconstructed data	ClAIN model performed the best
11	Kimura-Sandoval et al., Mexico, 2021	Multivariate logistic regression	-	To determine the risk of mortality/MV	Significant reduction in interpretation time, fast learning curve, increased objectivity	Small cohort. Only reconstructions with a slice thickness of 1.5 mm were analyzed	The model was successful
12	Parreco et al., USA, 2018	GBDT	-	To predict prolonged MV and tracheostomy placement	High specificity. The variable importance can be determined	Low sensitivity, information bias	The model was successful as it lead to improved outcomes by allowing for early intervention
			2. Prediction of weaning from MV and extubation				
13	Jia et al., UK, 2021	CNN	ANN, LR, Support Vector Machine, Decision Tree,	Predict weaning from MV based on	Explainability features like importance and	No risks of the model were described. Only	The CNN model performed the best

			Random Forest Tree	patient's information	counterfactual explanations	limitations of datasets and input variables were mentioned	
14	Liao et al., Taiwan, 2022	LR, RF, SVM, KNN, XGBoost, LightGBM, MLP	-	To predict time of weaning from MV	More precise decisions on weaning	Imaging was not analyzed	The prediction model of XGBoost showed the best testing results
15	Lin et al., Taiwan, 2021	XGBoost, RF, LR	-	To predict weaning from MV	The black-box issue has been largely mitigated	Not all features were reported	XGBoost/RF outperformed LR for predicting weaning outcomes in patients requiring prolonged MV
16	Pinto et al., Colombia/Spain, 2023	LDA, LR, backward feature selection, RFE, ANN, KNN, SVM	-	To analyze respiratory pattern variability	Many models were compared. Different techniques were combined	Clinical condition and comorbidities were not available. Bias	NN is more accurate than LDA The best classifier was SVM
17	Liu et al., Taiwan, 2022	LR, RF, SVM, KNN, lightGBM, XGBoost, MLP	-	To predict optimal timing for MV weaning	Many models were compared. Two-stage prediction approach	Small sample. Essential features and modalities were not included	The models were successful in predicting optimal time
18	Pai et al., Taiwan, 2022	XGBoost, CatBoost, LightGBM, RF, LR	-	To predict the timing of extubation	Many models were compared. High accuracy. The visualized interpretation of the model in the domain, feature and individual level	Single hospital database. Black box issue	Tree-based models like XGBoost, CatBoost, LightGBM, RF, performed the best
				3. Prediction of extubation failure			

19	Xia, China, 2022	LR, RF, KNN, SVM, XGBoost, LightGBM	-	To predict hypoxemia after extubation	Big dataset	Many clinical features were not available	The best models were the RF and LightGBM models
20	Hiremath et al., USA/China, 2021	C1AIN (CNN)	AIP, ACP	To predict MV use	Accurate, easy to deploy, hybrid approach	No access to the raw preconstructed data	ClAIN model performed the best
				4. Prediction of MV settings			
21	Tzavaras et al., Greece/UK, 2007	Fuzzy Neural (FUN) toolbox in MATLAB	-	Determine the RR and VT based on non-invasive parameters	Utilizes expert's knowledge for decision making. Can model complex systems	The difference between the predicted and actual outcome may be high	The method seems to provide good results for patients with four different COPD types
22	Radhakrishnan et al., India, 2022	Multilayer perceptron neural network	Human assessment	To predict level of inspired oxygen, MV mode and PEEP	Ability to integrate real time continuous pulse oximetry values with intermittent ABG readings	Small dataset	The model was good in prediction
23	Rees et al., Denmark, 2022	Clinical decision support system	-	To assess the need for changes in MV parameters	Reduces information volume, visuality	Not all parameters are analyzed	Visual patterns that explain the trade-offs necessary in MV can be recognized
24	Chen et al., China, 2022	Attention-based convolutional long short-term memory neural network	Rule-based, flow spectrum analysis, SVM, U-net, 3-layer 1 DCNN, 2-layer LSTM, model without optimization	To detect of patient-ventilator asynchrony from MV: ineffective expiration, double triggering	Combination of two models	Only two parameters of asynchrony were analyzed. Small sample. Clinical data were not considered	Combination of two models was successful in detection patient-ventilator asynchrony
25	Cheng, Taiwan, 2022	Lasso Regression, Ridge Regression, Elastic Net, RF,	-	To predict MV mode changes	Many models were compared	Time to extubation was not available	The models performed well

		XGBoost, SVM, and ANN					
26	Huang, China, 2022	LR, RF	-	To detect severe AKI in the first week of ICU	Big dataset. Long ICU observation time. Several ICUs. Internal and external validation	Only KDIGO-SCr criteria were used	The model performed well
27	Meena, India, 2022	LSTM and Bi-LSTM (RNN)	LR, RF, SVM, LGBM, MLP	To predict the pressure of MV	Many models were compared	Simulated (not real-life) experiment	Bi-LSTM performed the best
28	Pinto et al., Colombia/Spain, 2023	LDA, LR, backward feature selection, RFE, ANN, KNN, SVM	-	To analyze respiratory pattern variability	Many models were compared. Different techniques were combined	Clinical condition and comorbidities were not available. Bias	NN is more accurate than LDA The best classifier was SVM
29	Hagan et al., UK, 2020	AdaBoost, RandomForest, Bagging, ExtraTrees, GradientBoosting	LSTM	To predict over ventilation alerts in ICUs	Generalizable. Many models were compared	Some clinical features were not studied. Small sample	Boosted regression models performed the best
30	Perchiazzi et al., Sweden/Italy, 2003	Resilient back propagation (Rprop) (ANN)	MLF method	To estimate respiratory system compliance (CRS)	Robust, does not require explicit programming	Limited number of clinical conditions were considered	Rprop model was successful in extracting CRS without needing to stop inspiratory flow
				5. Prediction of MV outcome			
31	Gourdeau et al., Canada, 2022	Dense convolutional network	-	To predict MV outcome	Training on non-COVID patients	Small number of patients. Low quality of some imaging	The model was feasible in predicting COVID-19 mortality
32	Bermejo-Peláez, Spain, 2022	CNN	Radiomics. Radiologist	To predict clinical outcomes	Robust, high AUC score	The dataset had only axial slices. Small dataset	Model was successful in prediction

33	Maddux, USA, 2022	LR	-	To identify long-term morbidities and outcomes after prolonged MV	Broad pediatric population	Information about death was unavailable	The model performed well
6. Prediction of disease severity							
34	Han et al., China, 2022	Convolutional neural network and thresholding methods	Human assessment	To evaluate disease severity using chest CT	Saves time, predicts severity	AI model could not measure large and severely infiltrated lesions	AI might be utilized to assess the degree of illness in COVID-19 patients. GGOs had a higher predictive value for additional MV
35	Lu et al., China, 2021	Convolutional network	Clinical parameters alone	To predict the severity of disease in obese patients	-	Small sample. Some clinical parameters were not available	Obesity was related to severe CT lesions and outcomes. The model was successful
36	Bermejo-Peláez, Spain, 2022	CNN	Radiomics. Radiologist	To predict clinical outcomes	Robust, high AUC score	The dataset had only axial slices. Small dataset	Model was successful in prediction
37	Maddux, USA, 2022	LR	-	To identify long-term morbidities and outcomes after prolonged MV	Broad pediatric population	Information about death was unavailable	The model performed well
7. Mortality/survival prediction							
38	Mamandipoor et al., Italy/Spain/Austria/Germany/China/France/Argentina/Columbia/Canada/Mexico/Brazil/Uruguay/Australia/In	RNN - based LSTM	RF and LR	Predict mortality based on input variables and additional parameters	Direct processing of vector sequences; robustness, generalizability	The heterogeneity of data and averaging of ventilation parameters into a single daily value	The LSTM model outperformed the RF and LF models. Also, it is predicted that high granularity in a dataset can

	dia/Turkey/South Korea/Tunisia/Ecuador/Greece/Morocco/Thailand/USA, 2021						enhance the performance of AI
39	Aslam, Saudi Arabia, 2022	Model is not described - agnostic. Methods - SMOTE with oversampling and SMOTE with undersampling	-	To predict MV use and death	Big data sample	Some lab data are missing. Vaccination is not considered	The model was good in prediction
40	Greco, Italy, 2022	LR, balanced LR, RF	Uniform classifier	To predict survival at 7, 14, 21 and 28 days from ICU admission	Data-driven, automatic variable selection	Small sample. Comorbidities and physiological parameters were not fully available	Models were successful in predicting survival
41	Chan et al., Taiwan, 2022	XGBoost, RF, LR	-	To predict long-term mortality	Generalizable	TRL of the study is 4. Interpretability of the model is descriptive metric	XGBoost performed the best with high accuracy and illustrated distinct determinants
42	Bae et al., USA, 2021	U-Net DL model, Radiomics, CNN, LDA, QDA, RF	Radiologist Severity Scores, CNN, CNN and Radiomic-Map Embedding	To predict MV requirement	Validity on a multi-institutional dataset. Many models were compared	Nonuniform data. A limited number of clinical features were studied	The models were successful. The inclusion of radiomic features in DL improves model predictions
43	Kimura-Sandoval et al., Mexico, 2021	Multivariate logistic regression	-	To determine the risk of mortality/MV	Significant reduction in interpretation time, fast learning curve, increased objectivity	Small cohort. Only reconstructions with a slice thickness of 1.5 mm were analyzed	The model was successful

Search details
Search terms:
For the literature search, PubMed, Scopus, and the Cochrane Library were used. The search was narrowed with the help of search filters to find the most relevant articles.
Search terms: ("respiration, artificial" OR ("respiration" AND "artificial") OR "artificial respiration" OR ("mechanical" AND "ventilation") OR "mechanical ventilation") AND ("artificial intelligence" OR ("artificial" AND "intelligence") OR "artificial intelligence")
((((respiratory failure) AND (mechanical ventilation)) OR (artificial intelligence)) OR (machine learning)) OR (deep learning) Sort by: Most Recent (("respiratory insufficiency" OR ("respiratory" AND "insufficiency") OR "respiratory insufficiency" OR ("respiratory" AND "failure") OR "respiratory failure") AND ("respiration, artificial" OR ("respiration" AND "artificial") OR "artificial respiration" OR ("mechanical" AND "ventilation") OR "mechanical ventilation")) OR ("artificial intelligence" OR ("artificial" AND "intelligence") OR "artificial intelligence") OR ("machine learning" OR ("machine" AND "learning") OR "machine learning") OR ("deep learning" OR ("deep"AND "learning") OR "deep learning").

Search results
Search: (artificial ventilation) AND (artificial intelligence) Filters: Observational Study, Randomized Controlled Trial Sort by: Most Recent (("respiration, artificial"[MeSH Terms] OR ("respiration"[All Fields] AND "artificial"[All Fields]) OR "artificial respiration"[All Fields] OR ("artificial"[All Fields] AND "ventilation"[All Fields]) OR "artificial ventilation"[All Fields]) AND ("artificial intelligence"[MeSH Terms] OR ("artificial"[All Fields] AND "intelligence"[All Fields]) OR "artificial intelligence"[All Fields])) AND (observationalstudy[Filter] OR randomizedcontrolledtrial[Filter])
Results: 58
Search: (mechanical ventilation) AND (artificial intelligence) Filters: Observational Study, Randomized Controlled Trial Sort by: Most Recent (("respiration, artificial"[MeSH Terms] OR ("respiration"[All Fields] AND "artificial"[All Fields]) OR "artificial respiration"[All Fields] OR ("mechanical"[All Fields] AND "ventilation"[All Fields]) OR "mechanical ventilation"[All Fields]) AND ("artificial intelligence"[MeSH Terms] OR ("artificial"[All Fields] AND "intelligence"[All Fields]) OR "artificial intelligence"[All Fields])) AND (observationalstudy[Filter] OR randomizedcontrolledtrial[Filter])
Results: 56

Search: (artificial ventilation) AND (machine learning) Filters: Observational Study, Randomized Controlled Trial Sort by: Most Recent (("respiration, artificial"[MeSH Terms] OR ("respiration"[All Fields] AND "artificial"[All Fields]) OR "artificial respiration"[All Fields] OR ("artificial"[All Fields] AND "ventilation"[All Fields]) OR "artificial ventilation"[All Fields]) AND ("machine learning"[MeSH Terms] OR ("machine"[All Fields] AND "learning"[All Fields]) OR "machine learning"[All Fields])) AND (observationalstudy[Filter] OR randomizedcontrolledtrial[Filter])
Results: 34
Search: (artificial ventilation) AND (deep learning) Filters: Observational Study, Randomized Controlled Trial Sort by: Most Recent (("respiration, artificial"[MeSH Terms] OR ("respiration"[All Fields] AND "artificial"[All Fields]) OR "artificial respiration"[All Fields] OR ("artificial"[All Fields] AND "ventilation"[All Fields]) OR "artificial ventilation"[All Fields]) AND ("deep learning"[MeSH Terms] OR ("deep"[All Fields] AND "learning"[All Fields]) OR "deep learning"[All Fields])) AND (observationalstudy[Filter] OR randomizedcontrolledtrial[Filter])
Results: 4
